# The complete mitochondrial genome of the apple snail *Pomacea maculate* (Gastropoda: Ampullariidae)

**DOI:** 10.1080/23802359.2018.1511841

**Published:** 2018-10-26

**Authors:** Huirong Yang, Jia-En Zhang, Zhixin Deng, Hao Luo, Jing Guo, Chunxia Zhang, Yuanyuan Wu, Mingzhu Luo, Benliang Zhao

**Affiliations:** a College of Marine Sciences, South China Agricultural University, Guangzhou, China;; b Institute of Tropical and Subtropical Ecology, South China Agricultural University, Guangzhou, China;; c Key Laboratory of Agro-Environment in the Tropics, Ministry of Agriculture P.R. China, Guangzhou, China;; d Guangdong Engineering Research Center for Modern Eco-Agriculture and Circular Agriculture, Guangzhou, China

**Keywords:** Ampullariidae, *Pomacea maculate*, mitochondrial genome

## Abstract

We present the complete mitochondrial genome of *Pomacea maculate* in this study. The mitochondrial genome is 15,512 bp in length, containing 13 protein-coding genes, 2 rRNA genes, 22 tRNA genes. Overall nucleotide compositions of the light strand are 41.13% of A, 30.81% of T, 15.25% of C and 12.81% of G. Its gene arrangement and distribution are different from the typical vertebrates. The absence of D-loop is consistent with the Gastropoda, but at least one lengthy non-coding region is essential regulatory element for the initiation of transcription and replication. Phylogenetic tree is constructed by the maximum-likelihood method based on the complete mitochondrial genomes of 15 species of Caenogastropoda, using *Helix aspersa* as outgroup to assess their actual phylogenetic relationship and evolution. The result provides fundamental data for resolving phylogenetic and genetic problems related to effective management strategies.

*Pomacea maculata* (formerly *P. insularum*) (Perry, 1810), was introduced to Asia from Brazil and Argentina independently (Hayes et al. 2008). Subsequently, Lv et al. (2013) reported that *P. maculata*, was established in China. *Pomacea maculata* is one of two most common and highly invasive apple snail species as *P. canaliculata* (Lamarck, 1822) currently (Hayes et al. 2012), which strips vegetation, reproduces at tremendous rates, and have reduced rice production and caused ecosystem issues in Asia (Pimentel et al. 2005; Zedler and Kercher 2005; Burlakova et al. 2009). Some studies confirmed a much lower haplotype diversity of *P. maculata* in populations of China than that in their native countries Argentina and Brazil with a statistic ratio of 3:34 (Hayes et al. 2008; Lv et al. 2013; Yang et al. 2018). Furthermore, just a single lineage of *P. maculata* from Brazil was introduced into and established in China.

We sequenced its complete mitogenome to analyze phylogenetic relationship and evolutionary history for broadening the understanding of their population diversity, invasion processes and implementing effective management strategies. The specimen was sampled from Ningxi Teaching and Research Farm of South China Agricultural University in Guangzhou (E 113°29′, N 23°5′), and stored in the specimen museum of SCAU (accession number: 201502118).

The complete mitochondrial genome of *P. maculate* (Genbank accession number KY008699) is 15,512 bp in length, containing 13 protein-coding genes, 2 ribosomal RNA genes (rrns and rrnl), 22 transfer RNA genes (tRNA), which are encoded on the heavy strand except 8 tRNA genes (Met, Tyr, Cys, Trp, Gln, Gly, Glu and Thr) on the light strand. 22 tRNA genes vary from 64 to 75 bp in length, and all fold into the typical cloverleaf secondary structure. Among 13 protein-coding genes (total 11,238 bp) encoding 3,733 amino acids, the maximum is ND5 with 1,710 bp, and the minimum is ATP8 with only 159 bp. Rrns and rrnl genes are 934 and 1331 bp, respectively, located between the tRNA^Glu^ and tRNA^Leu^ genes and separated by the tRNA^Val^ gene. Overall nucleotide compositions of the light strand in descending order are 41.13% of A, 30.81% of T, 15.25% of C and 12.81% of G. Gene arrangement and distribution are different from the typical vertebrates (Yang, Sun, Zhao, Chen, et al. 2016; Yang, Sun, Zhao, Yang, et al. 2016; Yang, Xie, et al. 2016; Yang, Zhao, Sun, Chen, et al. 2016; Yang, Zhao, Sun, Xie, et al. 2016; Yang, Zhao, Sun, Yang, et al. 2016; Yang, Zhao, Sun, Zhang, et al. 2016; Yang, Zhao, Xie, et al. 2016), and similar to the Mollusca (Yang, Zhang, Deng, et al. 2016; Yang, Zhang, Guo, et al. 2016; Yang, Zhang, Luo, et al. 2016; Guo et al. 2017). The absence of D-loop is consistent with the Gastropoda (Yang, Zhang, Deng, et al. 2016; Yang, Zhang, Guo, et al. 2016; Yang, Zhang, Luo, et al. 2016; Guo et al. 2017), but at least one lengthy non-coding region is essential regulatory element for the initiation of transcription and replication (Wolstenholme 1992). There are 24 intergenic spacers (total 547 bp) varying from 3 to 141 bp in length, the largest of which is 141 bp between tRNA-Phe and NC III gene, and 2 gene overlaps (total 13 bp).

Phylogenetic tree is constructed by the maximum-likelihood method based on the complete mitogenomes of 15 species of Caenogastropoda, using *Helix aspersa* as outgroup to assess their actual phylogenetic relationship and evolution (Figure 1).

**Figure 1.**
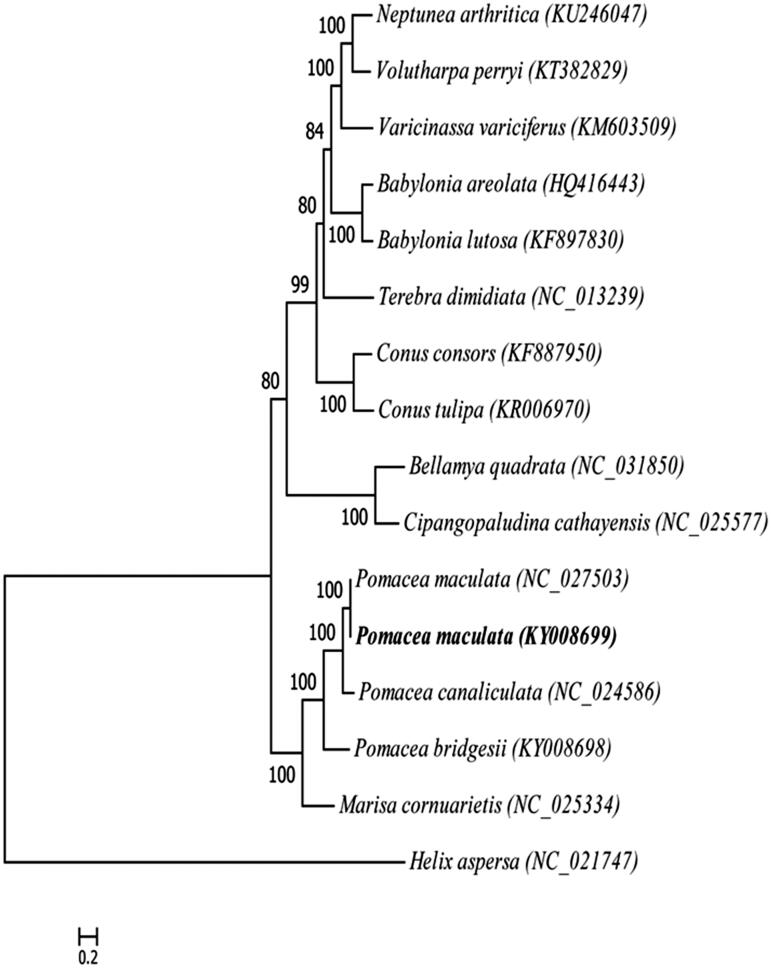
Phylogenetic tree generated by the maximum-likelihood method based on the complete mitochondrial genomes of 15 species of Caenogastropoda, using *Helix aspersa* as outgroup. The published sequences in GenBank adopted are *Neptunea arthritica* (KU246047), *Volutharpa perryi* (KT382829), *Varicinassa variciferus* (KM603509), *Babylonia areolata* (HQ416443), *Babylonia lutosa* (KF897830), *Terebra dimidiate* (NC_013239), *Conus consors* (KF887950), *Conus tulipa* (KR006970), *Bellamya quadrata* (NC_031850), *Cipangopaludina cathayensis* (NC_025577), *Pomacea maculate* (NC_027503), *Pomacea maculate* (KY008699), *Pomacea canaliculata* (NC_024586), *Pomacea bridgesii* (KY008698), *Marisa cornuarietis* (NC_025334), *Helix aspersa* (NC_021747).
